# The Role of Extracellular Vesicles in Bone Metastasis

**DOI:** 10.3390/ijms19041136

**Published:** 2018-04-10

**Authors:** Michela Rossi, Giulia Battafarano, Matteo D’Agostini, Andrea Del Fattore

**Affiliations:** 1 Bone Physiopathology Group, Multifactorial Disease and Complex Phenotype Research Area, Bambino Gesù Children’s Hospital, 00165 Rome, Italy; michela1.rossi@opbg.net (M.R.); giulia.battafarano@opbg.net (G.B.); 2 Clinical Laboratory, Bambino Gesù Children’s Hospital, 00165 Rome, Italy; matteo.dagostini@opbg.net

**Keywords:** bone metastasis, extracellular vesicles, exosomes

## Abstract

Multiple types of cancer have the specific ability to home to the bone microenvironment and cause metastatic lesions. Despite being the focus of intense investigation, the molecular and cellular mechanisms that regulate the metastasis of disseminated tumor cells still remain largely unknown. Bone metastases severely impact quality of life since they are associated with pain, fractures, and bone marrow aplasia. In this review, we will summarize the recent discoveries on the role of extracellular vesicles (EV) in the regulation of bone remodeling activity and bone metastasis occurrence. Indeed, it was shown that extracellular vesicles, including exosomes and microvesicles, released from tumor cells can modify the bone microenvironment, allowing the formation of osteolytic, osteosclerotic, and mixed mestastases. In turn, bone-derived EV can stimulate the proliferation of tumor cells. The inhibition of EV-mediated crosstalk between cancer and bone cells could represent a new therapeutic target for bone metastasis.

## 1. Introduction

Bone is the third most frequent site of metastasis, behind lung and liver. It is the preferential site for breast and prostate cancer [1,2]. Indeed, the relative incidence of bone metastasis is 70% for breast and prostate cancers, 60% for thyroid cancer, and 40% for bladder and lung tumors. Bone metastasis can dramatically impact quality of life, since patients may present with pain, hypercalcemia, fractures, and/or spinal cord compression, leading to neurological impairment and bone marrow aplasia. These skeletal related events (SRE) are associated with further complications, which negatively impact mobility, the ability to carry out daily tasks, quality of life, and patient mental state. Moreover, the median survival from diagnosis of bone metastasis is low, including 19–25% for breast cancer patients, 12–52% for prostate cancer patients, and about 6–9% for lung and bladder cancer patients [3].

Bone metastases are usually located in the most vascularized area of the skeleton; more than 80% occur in the vertebrae, ribs, and hips [2].

The metastatic process is a complex mechanism that requires local tumor cell growth and induction of angiogenesis, tumor cell detachment from the primary site and invasion, entry into the systemic or lymphatic vasculature, arrest in the capillary bed of the bone, exit of tumor cells from the circulation (extravasion), colonization, and proliferation at the distal sites [4]. The particular attraction of cancer cells for a target organ was originally described by Paget, who proposed the theory of “seed and soil”, emphasizing the importance of the host milieu in the selectivity of tumor cells to metastasize. This process requires interactions among the tumor cells (seed), the circulatory system, and the host microenvironment (soil) [5,6].

According to Paget theory, cancer cells are able to migrate and colonize environments that allow for their proliferation and survival by the production of growth factors and cytokines, expression of specific receptors, and stroma features [5,6]. However, this theory does not completely explain the reasons why a tumor derived from paired organs does not commonly colonize the contralateral organs. Ewing proposed that the choice of which organs to colonize is simply associated with mechanical and anatomic reasons; according to this theory, the first organ the tumors cells pass through via blood circulation is the preferential site for metastasis [7]. However, a large volume of blood from the breast reaches the heart and spleen, and in these sites breast cancer rarely metastasizes [8].

The proliferation of cancer cells in the bone is a complex mechanism, by which tumor cells adapt and grow in a foreign microenvironment, assuming some features of the bone cells in an “osteomimecry” process and destroying the bone virtuous cycle [9]. In an adult of normal physiological condition, the bone resorption activity of osteoclasts is balanced with the bone formation activity of osteoblasts [10]. Particularly, osteoclasts are multinucleated cells of hematopoietic origin that differentiate from mononuclear precursors cells under the influence of M-CSF (Macrophage Colony-Stimulating Factor) and RANK-L (Receptor Activator of Nuclear factor kappa-B Ligand), that are secreted by stromal cells, osteoblasts, osteocytes, and immune cells [10]. Signals generated by the binding of M-CSF to the cell-surface receptor colony-stimulating factor-1 receptor (c-fms) of osteoclast precursors induce the proliferation and expression of the receptor RANK, leading to osteoclast differentiation. The interaction between RANK and RANK-L stimulates the fusion of osteoclast precursors to generate mature multinucleated cells and promotes the survival and activity of osteoclasts [11,12].

During bone resorption activity, osteoclasts release Transforming Growth Factor beta (TGF-β), Fibroblast Growth Factor (FGF), Insulin Growth Factor (IGF), Platelet-Derived Growth Factor (PDGF), and Bone Morphogenetic Proteins (BMP) that are trapped in the bone matrix [10]. These factors are able to stimulate the differentiation of mesenchymal stem/stromal cells into osteoblasts and the deposition of bone matrix. Moreover, osteoclasts are able to secrete factors named “osteoclastokines”, that are able to influence osteoblast differentiation and activity in a bone resorption-independent mechanism [13]. Some of these secreted osteoclast factors were recently discovered, including Platelet Derived Growth Factor (PDGF) BB [14], Hepatocyte Growth Factor (HGF) [15], and Sphingosine 1 Phosphate (S1P) [16]. We have shown in vitro that TRAcP (Tartrate Resistant Acid Phosphatase) secreted by osteoclasts could act as an ATPase after proteolitic cleavage by trypsin and cathepsin K, and stimulate osteoblasts, inducing an increase in ALP (Alkaline Phosphatase) activity [17]. In turn, osteoblasts regulate osteoclastogenesis by the release of M-CSF, RANK-L, and RANK-L receptor decoy Osteoprotegerin (OPG) [10].

**Figure 1:**
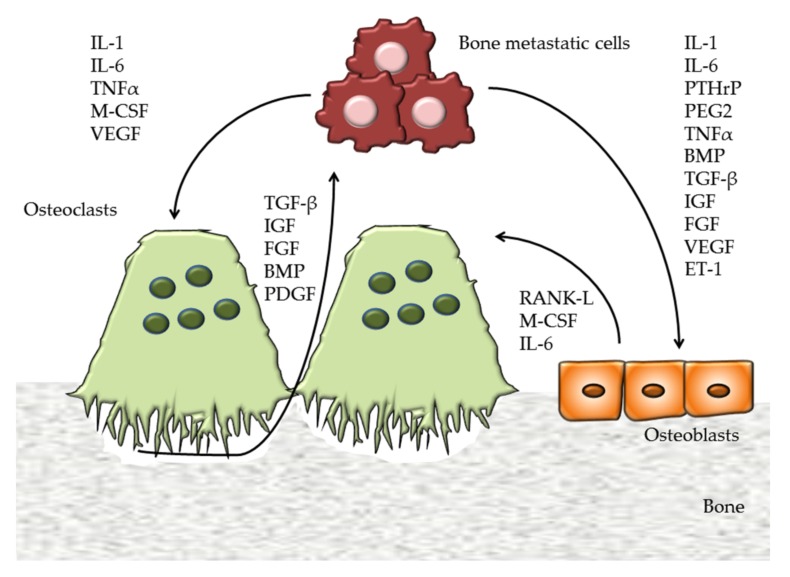
Vicious cycle of bone metastasis. Tumor cells destroy the virtuous cycle of bone remodeling stimulating osteoclast differentiation and activity by the secretion of IL-1 (Interleukin 1), IL-6 (Interleukin 6), TNFα (Tumor Necrosis Factor alpha), M-CSF (Macrophage Colony Stimulating Factor), and VEGF (Vascular Endothelial Growth Factor). TGF-β (Transforming Growth Factor beta), IGF (Insulin Growth Factor), FGF (Fibroblast Growth Factor), BMP (Bone Morphogenetic Protein), and PDGF (Platelet Derived Growth Factor) are released from the bone matrix during bone resorption and stimulate tumor cells. Bone metastastic cells influence osteoblast activity by IL-1, IL-6, PTHrP (Parathyroid hormone-related protein), PEG2 (Prostaglandin E2), TNFα, ET-1 (Endothelin-1), BMP, and various growth factors. In turn, osteoblasts secrete factors that stimulate osteoclastogenesis, including RANK-L (Receptor activator of nuclear factor kappa-B ligand), M-CSF, and IL-6.

Tumor cells can mimic bone cells generating a vicious cycle (Figure 1). Breast cancer cells resident in bone metastases are characterized by high levels of cathepsin K [18,19] and Parathyroid hormone-related protein (PTHrP), stimulating RANK-L expression in osteoblasts enhancing osteolysis [20].

The metastatic cells alter the bone remodeling process, defining osteolytic, osteosclerotic, and mixed lesions [21]. In osteolytic metastasis produced by a large majority of breast cancer cells, the osteoclast activity is stimulated by cancer cells that release interleukin-1 (IL-1), IL-6, PTHrP, prostaglandin E2 (PEG2), TNFalpha (Tumor Necrosis Factor), VEGF (Vascular Endothelial Growth Factor), and M-CSF (Figure 1). Particularly, these factors stimulate osteoclast differentiation and activity and inhibit bone formation by osteoblasts. This mechanism allows tumor cells to grow inside the bone and continue to proliferate, destroying the bone burdens [22].

In the osteosclerotic bone metastasis as in prostate cancer, bone formation also occurs. This process is partly due to a direct stimulation of osteoblasts, or inhibition of osteoclasts. Prostate cancer cells express endothelin-1 (ET-1) that binds its receptor (ETR) on the osteoblasts, stimulating Wnt signaling [23]. Moreover, it was shown that Prostate-Specific Antigen (PSA) is able to cleave PTHrP, reducing bone resorption and allowing bone formation to predominate [24]. Although a substantial body of evidence established that osteoclast activation is required for osteosclerotic metastases [25], this mechanism is not totally known.

In mixed bone metastasis, cancer cells are able to stimulate osteoclastogenesis and bone formation, thus, patients can have both osteolytic and osteosclerotic lesions [21].

At present, the use of antiresorptive drugs (bone-targeted agents; BTAs) represents the most effective treatment for patients with bone metastases, to decrease SRE. Bisphoshonates are analogues of pyrophosphate and have a great affinity for the bone matrix. During bone resorption activity, they induce osteoclast apoptosis. They are used to arrest hypercalcemia and limit tumor growth [26]. Denosumab is a fully human IgG2 monoclonal antibody that inhibits RANK-L, arresting osteoclast differentiation. Stopeck et al. showed that the suppression of bone turnover markers is greater in patients treated with Denosumab than in those treated with bisphosphonates [27]. Several in vitro studies with bisphosphonates, such as pamidronate and zoledronic acid, revealed inhibition of the mevalonate pathway in breast and prostate cancer cells, which led to the induction of the apoptosis [3]. Moreover, pre-clinical evidence suggests that the inhibition of RANK-L inhibits tumor proliferation, reducing skeletal tumor burden and visceral metastasis in a direct manner [28]. Other treatments for bone metastasis are radiotherapy, ablation, and surgery for fractures and peripheral nerve compression [9]. The choice of which treatment to use is related to several parameters, including symptoms, state of health, the localized or diffuse presence of metastasis and the feature of primary tumors.

## 2. Extracellular Vesicles

**Figure 2:**
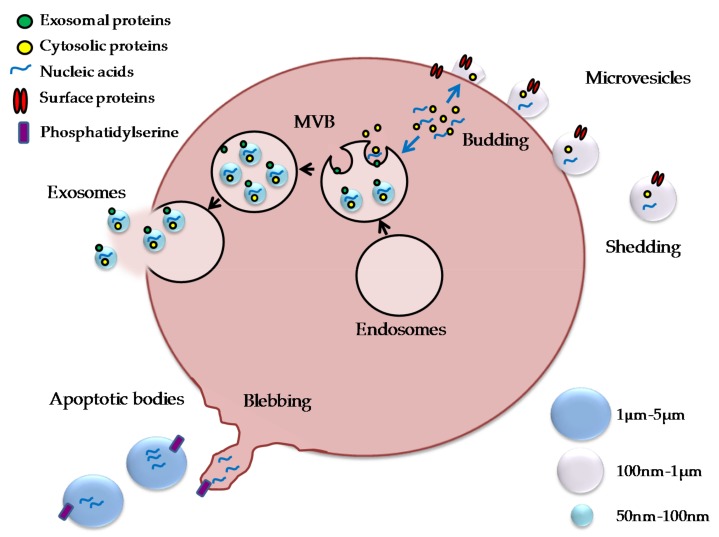
Extracellular vesicles. Exosomes, microvesicles, and apoptotic bodies differ in dimensions and biogenesis. They carry several molecular components, including cytosolic and membrane proteins, receptors, and nucleic acids. Exosomes are small vesicles (50–100 nm) that are derived from multivesicular bodies (MVB), and are released by fusion of MVB with the cell membrane; microvesicles are larger vesicles (100 nm–1 µm) and are derived from budding of the plasma membrane; apoptotic vesicles have a size ranging from 1 to 5 µm, and are released by the blebbing process during apoptosis.

The extracellular space of the multicellular organism represents a complex environment, composed of metabolites, ions, proteins, and polysaccharides. It is now well established that it contains membrane-limited vesicles, which play important roles in cell-to-cell communication. Extracellular vesicles (EV) represent a heterogeneous population of membrane-limited vesicles characterized by exosomes, microvesicles, and apoptotic bodies [29]. Although initially met with skepticism, their presence is now well established as they represent an important means of cellular crosstalk [30]. Indeed, it was shown that cells by vesicles could share proteins, nucleic acids, and intact organelles, such as mitochondria [31]. The different types of vesicles differ in their size and biogenesis [32] (Figure 2).

The exosomes are small rounded structures with a size of approximately 50–100 nm in diameter, overlapping the dimensions of some viruses. They were described for the first time by Trams et al. as exfoliated vesicles with ectoenzyme activity [33]. They are released constitutively and under specific stimuli, and derive from the fusion of multivesicular bodies (MVB) with the plasma membrane. MVB are formed during the maturation of early endosomes into late endosomes, due to the accumulation of intraluminal vesicles [34]. They display phoshatidylserine on the outer membrane and markers such as CD63, CD81, CD9, TSG10 (Tumour Susceptibility Gene 10), and LAMP1 (Lysosomal-Associated Membrane Protein 1) [30].

Microvesicles are membrane vesicles with a diameter ranging from 100 nm to 1 μm. They were described for the first time by Chargaff and West in 1946 as a precipitable factor in platelet-free plasma [35]. They came from budding of the plasma membrane. The biogenesis of microvesicles requires modification within the plasma membrane, including changes in the lipid components and proteins, and alteration of Ca^2+^ levels. Tranlocases such as flipplase and floppase, calpain and scramblase are essential for the rearrangements of membrane phospholipids and alteration of the cytoskeleton, leading to budding and microvesicle formation [29]. Characterization studies suggest that the microvesicles’ population is composed of vesicles either with or without phosphatidylserine externalization [36].

Apoptotic vesicles are 1–5 μm in diameter and are released as blebs of cells undergoing apoptosis. They are characterized by phosphatidylserine externalization and can contain DNA fragments and RNA. Apoptotic bodies have been investigated, particularly in the context of communication between cancer cells, providing evidence that they can also carry RNA to neighboring cells [37].

These extracellular vesicles represent a cargo of vesicles containing protein and nucleic acid, including DNA, mRNA, and miRNA [38]. Extracellular vesicles in the proximity of secreting cells may enter biological fluids, such as plasma or urine, among many others, and act as endocrine-like messengers, potentially mediating numerous biological processes. They can stimulate a target cell in different ways: (1) adhesion of EV to the recipient cell surface through lipids, or ligand–receptor interaction and stimulation of inhibition of intracellular pathways; (2) fusion of the vesicles with the target cells and release of its content in the cytoplasm; (3) internalization of the vesicles by target cells through mechanisms such as endocytosis [39].

EV are released from many cells, including mesenchymal/stromal cells, epithelial cells, immune cells, bone, and tumor cells [34].

Many studies were published showing their exciting effects on cell communication. Extracellular vesicles are involved in cell plasticity, tissue regeneration, regulation of the nervous system, and immune response [30]. Indeed, extracellular vesicles released by mesenchymal stem/stromal cells (MSC) can have immunomodulatory effects, which stimulate the T regulatory CD4^+^/CD25^+^/CD127^low^ population [40]. We previously showed that EV can exert different effects on the tumor cells. We demonstrated that vesicles isolated from the conditioned medium of the MSC from the umbilical cord and bone marrow were able to inhibit the proliferation of glioma cells, whereas, those derived from adipose tissue stimulated proliferation [41]. Interestingly, EV secreted from placenta seem to contribute to fetomaternal tolerance, reducing CD3ζ expression by T cells [42], and the cytotoxicity of NK and CD8+ T cells [43].

Exosomes and microvesicles also represent a mechanism used by cells to protect against internal and external stress (e.g., release of caspase 3, release of complement C5b-9 complex against lysis) [44]. Boing et al. suggested that cells use vesicles to remove dangerous or potentially dangerous molecules that may threaten the cells’ viability and survival. By efficient packaging of such molecules into vesicles, the cells can remain healthy and viable. In this manner, extracellular vesicles contribute to cellular homeostasis by functioning as garbage bags which can then be phagocytosed and degraded by other cells [44]. Indeed, EV are used by tumor cells to avoid apoptosis and to “transform” cells [45].

### 2.1. Exosome in Bone and in Bone Metastatic Cells

#### 2.1.1. EV in Bone

The bone marrow is a complex compartment where the communication between cells is mediated by cell–cell contact and soluble factors controlling bone remodeling activity. Recent studies suggest that extracellular vesicles represent a tool used by bone cells to regulate skeletal activities. Osteoclasts and osteoblasts are able to stimulate each other, not only by direct cell–cell interaction and soluble factors, but also by the release of extracellular vesicles [46].

Protein characterization of EV isolated from osteoclasts revealed that they express TSG101, heat shock protein (HSP) 70, β-actin, cell differentiation (CD) 63, and the epithelial cell adhesion molecule (EpCAM) [47,48].

Sun W et al. demonstrated that exosomes isolated from the conditioned medium of an osteoclast culture were able to be internalized by osteoblasts and inhibit their function by transfer of miR-214 [48]. The exosome recognition by osteoblasts is mediated by EphrinA; EphrinA2 acts as a “coupling inhibitor”, since EphrinA2 reverse signaling into osteoclasts enhances their differentiation, and EphA2 forward signaling into osteoblasts suppresses bone formation and mineralization [49]. Moreover, it was shown that EV isolated from monocyte cultures were able to induce osteoblast differentiation in vitro [50]. In vivo experiments with exosomes derived from osteoclast cultures confirmed the ability of these EV to fuse with osteoblasts. Li D et al. showed that osteoclast exosomes containing miR-214-3p inhibit the osteoblast function [47]. The levels of this miRNA were found to be increased in the serum of elderly women and in ovariectomized mice [47]. miR-214-3p transferred from osteoclasts to osteoblasts inhibits osteoblast differentiation and activity, reducing the expression of Atf4 (Activating transcription factor 4), Alp (Alkaline phosphatase), Bglap (bone gamma-carboxyglutamic acid-containing protein, osteocalcin) and Col1α1 (collagene type 1 alpha 1) [48]. The exosomes released by osteoclast precursors and mature cells have different effects on osteoclast lineage. It was demonstrated that EV isolated from osteoclast precursors were able to induce osteoclast differentiation, while those derived from mature osteoclasts formed fewer osteoclasts. Huynh et al. showed that RANK levels were higher in osteoclast-derived exosomes than in precursor-EV. This observation suggests that osteoclast-derived EV could entrap RANK-L, and thus, limit osteoclastogenesis [51].

Mesenchymal stem/stromal cells and osteoblasts secrete exosomes too. It was shown that exosomes derived from mesenchymal stem cells can be involved in bone remodeling since they induce osteoblast differentiation in autocrine and paracrine mechanisms. Vallabhaneni et al. characterized the total cargo of EV isolated from mesenchymal stem cells, and identified tumor-supportive miRNAs including miR-21 and miR-34a, proteins such as PDGFR-β, TIMP-1 (Tissue inhibitor of matrix metalloproteinases 1), and TIMP-2, bioactive lipids and metabolites [52]. In vivo experiments of bone regeneration established that the treatment of calvarial bone defects with MSC-EV promoted bone repair. miR-196, miR-27a, and miR-206 are particularly enriched in exosomes isolated from bone marrow mesenchymal stem cells and can induce osteogenic differentiation [53]. EIF2 (eukaryotic initiation factor) in exosomes of osteoblasts stimulate osteogenic differentiation of MSC [54]; indeed, EIF2 is involved in bone morphogenetic protein (BMP)-2-induced osteoblast differentiation, playing a central role in the development of the skeletal system [55,56]. Extracellular vesicles isolated from the conditioned medium of a mature osteoblast cell line, MC3T3-E1, stimulated the osteoblast differentiation of osteoblast precursor ST2, altering the miRNA profile towards pathways that play pivotal roles in the differentiation (Wnt signaling pathway, insulin signaling pathway, and TGF-β signaling pathway) and function (calcium signaling pathway) of osteoblasts [57]. Hwang et al. reported that miR-140-3p, which is highly expressed in osteoblast exosomes, affects osteoblast differentiation by inhibiting BMP2 expression [58].

Moreover, it was shown that PTH stimulates RANK-L expression in osteoblast exosomes, and Delg et al. attested that RANK-L-rich exosomes are able to stimulate osteoclast differentiation, inducing the nuclear translocation of nuclear factor of activated T cells (NFATc1) [59]. RANK-L positive extracellular vesicles could induce osteoclastogenesis by binding directly to osteoclast precursors or fusing with stromal cells to increase the amount of RANK-L. It was demonstrated that EV internalization occurs when the specific recognition between osteoblast-derived vesicles and target cells cannot be achieved [59]. Solberg et al. showed that LAMP-1 (lysosomal membrane protein 1)-positive EV isolated from rat osteoblasts and osteocytes contain RANK-L, OPG, and TRAcP [60]. Mineralized osteoblasts can secrete exosomes containing miR-503-3p, which is able to inhibit osteoclast differentiation reducing RANK expression [61].

Moreover, it was shown that osteocytes secrete vesicles regulating bone remodeling; indeed, osteocyte exosomes containing miR-218 can mediate the downregulation of sclerostin, thus stimulating osteoblast differentiation. Sato et al. suggested that exosomes derived from osteocytes could act in the bone microenvironment, or could be released into circulation [62].

#### 2.1.2. Exosomes in Bone Metastasis

Recent studies suggest that exosomes derived from primary tumors could facilitate the initial communication between the primary tumor and site of metastasis. The secretion of exosomes and their ability to reach a distal site can modify the distal microenvironment, and can recall primary tumor cells, allowing for their proliferation [63,64]. It was shown that tumor cells exposed to hypoxia released exosomes with angiogenic metastatic potential [65].

Regarding bone metastasis, exosomes are important for the bidirectional stimulation of tumor cells and bone cells. Tumor cells are able to destroy the virtuous cycle of bone remodeling by the secretion of extracellular vesicles that can participate in bone lesion onset [45].

Valencia et al. [66] showed that EV isolated from the miR-192 overexpressing metastatic human adenocarcinoma A549 cell line, could reduce an in vivo osteolytic lesion and act in a tumor cell-autonomous manner by suppressing gene expression in tumor cells, modulating invasiveness, and metalloprotease activities. The treatment of endothelial cells with miR-192 abrogates the angiogenic program by repression of proangiogenic IL-8, ICAM (Intercellular Adhesion Molecule 1), and CXCL1. These data support the hypothesis that the isolated EV could condition the bone compartment, creating a conducive microenvironment that deranges osteolytic lesions and bone colonization, presumably by inhibiting angiogenesis in vivo [66].

Tumor-derived extracellular vesicles are able to directly influence bone cells. Karlsoon et al. showed that the murine prostate cancer line TRAMP-C1 inhibits the proliferation of osteoclast precursors, as well as the differentiation of bone-derived osteoclast precursors, and the differentiation of the monocytic cell line RAW264.7 into osteoclasts. Particularly, it was shown that RAW264.7 cells are able to internalize exosomes in 1 h, and that treatment induces a reduction in *dc-stamp* (Dendritic Cell-Specific Transmembrane Protein), *tracp*, *cathk* (Cathepsin K), and *mmp9* (Matrix metallopeptidase 9) expression [67]. This data can explain the reason why prostate cancer mainly exhibits osteosclerotic bone metastasis, while inhibiting osteoclast function.

Taverna et al. [68] evidenced that non-small cell lung cancer (NSCLC) cells secrete exosomes containing the EGFR (epidermal growth factor receptor) ligand and Amphiregulin (AREG); these extracellular vesicles are able to induce the in vitro osteoclast differentiation of murine RAW264.7 cells by activation of EGFR phosphorylation, and the induction of *mmp-9* and *tracp* expression. The relevance of this data was supported by the fact that in NSCLC patients AREG plasma levels were correlated with poor prognoses and that patient exosomes were able to modulate osteoclastogenesis of human osteoclast precursors. AREG knockdown, neutralizing the antibodies of AREG, and co-treatment with NSCLC-exosomes and epidermal growth factor receptor–tyrosine kinase inhibitor Erlotinib revert the osteoclast differentiation induced by exosomes [68].

Moreover, exosomes released by multiple myeloma cells increase the viability and migration of osteoclast precursors, through the increasing of CXCR4 (C-X-C chemokine receptor type 4) expression and differentiation. Additionally, these exosomes stimulate the activity of mature cells and the expression of *TRAcP* and *MMP9*. Multiple myeloma cell-derived exosomes labeled with PKH-26 were internalized by the murine macrophage cell line RAW264.7, and after incubation of 3 h displayed typical perinuclear localization. Similar results were obtained with extracellular vesicles contained in multiple myeloma patients’ sera [69]. Inder et al. showed that extracellular vesicles isolated from PC3 culture medium are internalized into osteoclast precursors and osteoblasts, stimulating osteoclastogenesis 37-fold and osteoblast proliferation 1.5-fold, respectively; this effect was lost in PC3 cells transfected with cavin-1 (also known as polymerase I and transcript release factor; PTRF) vector. The authors demonstrated that cav-1 was not present in the vesicles, revealing an indirect mechanism of action. It was shown that its expression alters EV cargo selection rather than morphology, size, and quantity of the released EV [70].

Regarding the ability of tumor cells to affect osteoblast differentiation and activity, it was shown that exosomes isolated from MDA PCa 2b cells (a PCa bone metastasis cell line) contain miR-141-3p and can transfer their content into osteoblasts; miR-141-3p reduced the protein levels of its target gene *DLC1* and activated p38 MAPK signaling, which increased the expression of *OPG* and further promoted osteoblast activity [71].

Recently, Hashimoto et al. performed a comprehensive expression analysis of miRNAs released by several human cell lines and identified a cluster of eight miRNAs in exosomes from prostate cancer that induce osteosclerotic lesions. Particularly, cancer exosomal miR-940 was able to induce the osteogenic differentiation of mesenchymal stem cells, by targeting *ARHGAP1* (Rho GTPase Activating Protein 1) and *FAM134A* (Family With Sequence Similarity 134 Member A). The relevance of the selected miRNA to induce osteosclerotic lesions was confirmed by the fact that its overexpression in the osteolytic phenotype-inducing cancer line MDA-MB-231 induced extensive osteoblastic lesions [72]. This study provides a demonstration of a cancer-secreted miRNA-induced osteoblastic-type bone metastasis serving as an osteotropic factor in the bone microenvironment.

If aforementioned studies suggest that tumors can secrete EV capable of altering bone remodeling activity, it was demonstrated that bone cells are able to secrete vesicles that affect cancer cells. Indeed, it was shown that exosomes from bone marrow mesenchymal stem cells could promote dormancy of human breast cancer cells in bone marrow. MSC primed by cancer cells can display a distinct profile (in terms of miRNA) of exosomes compared to naïve cells, which can favor their survival and dormancy. In vivo targeting of miR-222-223 reversed the quiescent phase of breast cancer cells into chemosensitive cells [73]. Regarding the effects on MSC-derived EV on the growth of cancer cells, contrasting results have been published, since exosomes and microvesicles can stimulate or inhibit the proliferation of tumor cells. This discrepancy could be related to the experimental protocol. Particularly, for in vivo experiments, the timing of injection is critical; if EV are administered simultaneously to the administration of tumor cells they promote tumor growth; when EV are injected after the establishment of the tumor, they exert an antiproliferative effect [74].

Extracellular vesicles isolated from human osteoblasts can promote PC3 cells in vitro. Recently, Morhayim et al. treated PC3 cells with exosomes derived from non-mineralizing (NMOB) and mineralizing osteoblasts [75]. They showed that the fluorescence intensity of PC3 cells treated with marked MOB-EV was higher than that observed in cells treated with marked NMOB-EV. Given this difference in the possible way of internalization, both types of EV stimulated the proliferation of PC3 cells and the expression of genes involved in cell survival and growth [75].

Exosomes released by osteoclasts can stimulate tumor proliferation, as miR-378 promotes tumor growth, angiogenesis, and tumor cell survival through the repression of tumor suppressors SuFu and Fus-1 [76]. Moreover, these cells release miR-21, which stimulates tumor cell proliferation [77].

## 3. Conclusions

The development of bone metastases is a complex process involving crosstalk between disseminated cancer cells and the bone microenvironment. Bone metastases are largely incurable and associated with significant morbidity that negatively impacts quality of life in metastatic cancer patients.

The discovery of extracellular vesicles in body fluids opened new frontiers about the possibility of promptly identifying the presence of tumors, reducing metastatic events, and identifying new therapeutic approaches.

The presence of tumor extracellular vesicles in the circulation could represent the means by which tumor cells choose the distal site of proliferation. Tumor-secreted extracellular vesicles can influence the distal microenvironment, modifying the resident cells to attract cancer cells and initiate a metastatic event. However, the trafficking events that govern the release of extracellular vesicles from bone and tumor cells as well as the uptake by the recipient cells remain largely unknown.

Researchers in this area are developing techniques to isolate disease-associated vesicles to define their selective cargo and improve their sensitivity as biomarkers.

The procedure for their isolation should be optimized, since many protocols are used, including differential ultracentrifugation, flotation on density gradients, polyethylene glycol precipitation, polymer-based precipitation, and immunoprecipitation. The use of several protocols allows the isolation of different populations of extracellular vesicles and a standardization of the procedure is necessary to reveal alterations of the vesicle set in the progression of a disease.

Regarding bone metastasis, the bone-derived exosomes could be an important tool to vehicle drugs into bone in order to counteract the metastasis and restore the normal bone remodeling activity. The EV-based therapeutic approach has already been tested. It has already been established that extracellular vesicles secreted by dendritic cells stimulate the immune system and can be used as antitumor vaccines [78].

Bruno et al. demonstrated that in vivo intra-tumor administration of microvesicles in established tumors generated by subcutaneous injection of HepG2 hepatoma, Kaposi’s sarcoma, and Skov-3 ovarian tumor cell lines in severe combined immunodeficiency (SCID) mice significantly inhibited tumor growth [79].

In our previous work, we displayed that vesicles isolated from MSC (MSC-EV) can be charged with chemotherapeutic drugs, including vincristine [41]. Loading the drug into EV resulted in a two-fold increase of cytotoxicity when compared to the free compound; this effect is likely due to facilitated intracellular drug delivery. Indeed, we showed that the association of MSC-EV with tumor cells takes place after 1 h, reaching complete internalization after 24 h [41]. This study also confirmed that MSC-EV can function as Trojan horses to deliver antiblastic drugs into cancer cells. Moreover, since our unpublished results gave evidence that EV isolated from MSC can reach the tumor, EV could be used as a therapeutic approach to vehicle drugs, specifically in tumor cells, reducing the side effects associated with conventional therapy. Li et al. suggestedd that exosomes isolated from osteoclasts can reach preferentiality in bone after in vivo injections [47]. These features of exosomes make them good candidates to vehicle antitumoral, or antiresorptive, or bone formation stimulating drugs into bone lesions.

One of the biggest challenges for the future is to produce extracellular vesicles in large scale for clinical application, which also underlines the development of methods for validation and quantification of vesicles. Despite the enormous therapeutic potential, the field of extracellular vesicles still requires further pre-clinical and clinical studies to translate exosomes from bench to bedside. Clearly, research on EV biodistribution and toxicity is needed to better define the therapeutic potential of these nanoparticles. Cell biologists and physicians working side by side in a complementary manner will certainly shed further light on the field of vesicles, identifying new therapeutic approaches for bone metastases and bone diseases.
